# 

**Published:** 1883-06

**Authors:** 


					﻿A LIBERAL OFFER.
To any person who will send $1.25, we will mail the Indepen-
dent Practitioner for the balance of the year, commencing with
the June number. We will not agree however, that this offer shall
stand open longer than until July 15th prox.
Contributors:
Alf. L. Loomis, M.D.................New	York
Fessenden N. Otis, M.D..............
F. H. Bosworth, M.D.................
<J. L. Little, 31.D..................  “
J. Williston Wright, M.D.............. “
C. E. Francis, D.D.S., M.D.S.......... “
A. J. C. Skene, M.D...........Brooklyn,	N. Y.
C. A. Marvin, D.D.S...........
1 Edwin T Darby. M.D., D.D.S........Phila.,	Pa.
1 P. S. Wales, M.D........Surg.-Gen’l U. S. Navy
I II. F. Campbell, M.D............Augusta, Ga.
i C. II. Mastin, M.D., LL.D........Mobile, Ala.
J. J. Chisolm, M. D,..........Baltimore, Md.
C. F. Bevan, M.D..............
I.	E. Atkinson, M.D............... “
H. McGuire, M.D................Richmond,	Va.
F. P. Porcher, M.D..........Charleston,	S. C.
Jas. T. Whittaker, M.D........Cincinnati,	O.
J.	Ransohoff, M.D., F.R.C.S....	“
F. Forchheimer, M.D............... •*
A. R. Jackson, M.D..............Chicago,	Ill.
S. V. Clevenger. M.D............... “
E. F. Ingals, M.D.................. “
J. H. Carstens, M.D...........Detroit,	Mich.
PROSPECTUS
Fob Vol. IV., 1883, of the Independent
Pbactitioneb.
This Journal is independent of colleges,
cliques, isms or any advertising firm; there-
fore impartiality pervades its columns in the
sole interest of the Profession.
It is cosmopolitan in its circulation and
scientific resources; consequently of equal
interest to the specialist and general prac-
titioner everywhere.
That which is new and useful in Medicine.
Surgery, Obstetrics, Dentistry, Pathology
and Popular Science is given to its readers
in an intelligent and concise style.
				

